# The potential role of advanced glycation end products (AGEs) and soluble receptors for AGEs (sRAGE) in the pathogenesis of adult-onset still’s disease

**DOI:** 10.1186/s12891-015-0569-3

**Published:** 2015-05-09

**Authors:** Der-Yuan Chen, Yi-Ming Chen, Chi-Chen Lin, Chia-Wei Hsieh, Yen-Ching Wu, Wei-Ting Hung, Hsin-Hua Chen, Joung-Liang Lan

**Affiliations:** Faculty of Medicine, National Yang-Ming University, Taipei, Taiwan; Department of Medical Education and Research, Taichung Veterans General Hospital, Taichung, Taiwan; Institute of Biomedical Science, National Chung-Hsing University, Taichung, Taiwan; Institute of Microbiology and Immunology, Chung-Shan Medical University, Taichung, Taiwan; Division of Allergy, Immunology and Rheumatology, Taichung Veterans General Hospital, Taichung, Taiwan; Division of Immunology and Rheumatology, China Medical University Hospital, Taichung, Taiwan; Division of Allergy, Immunology and Rheumatology, Department of Internal Medicine, Taichung Veterans General Hospital, No. 160, Sec. 3, Chung-Kang Rd., Taichung, 407 Taiwan

**Keywords:** Advanced glycation end products (AGEs), Soluble receptor for AGEs (sRAGE), Pathogenesis, Adult-onset Still’s disease (AOSD), Systemic lupus erythematosus (SLE)

## Abstract

**Background:**

Accumulating evidence has demonstrated a pathogenic role of advanced glycation end products (AGEs) and receptors for AGEs (RAGE) in inflammation. Soluble RAGE (sRAGE), with the same ligands-binding capacity as full-length RAGE, acts as a “decoy” receptor. However, there has been scanty data regarding AGEs and sRAGE in adult-onset Still’s disease (AOSD). This study aimed to investigate AGEs and sRAGE levels in AOSD patients and examine their association with clinical characteristics.

**Methods:**

Using ELISA, plasma levels of AGEs and sRAGE were determined in 52 AOSD patients, 36 systemic lupus erythematosus(SLE) patients and 16 healthy controls(HC). Their associations with activity parameters and disease courses were evaluated.

**Results:**

Significantly higher median levels of AGEs were observed in active AOSD patients (16.75 pg/ml) and active SLE patients (14.80 pg/ml) than those in HC (9.80 pg/ml, both p < 0.001). AGEs levels were positively correlated with activity scores (r = 0.836, p < 0.001), ferritin levels (r = 0.372, p < 0.05) and CRP levels (r = 0.396, p < 0.005) in AOSD patients. Conversely, significantly lower median levels of sRAGE were observed in active AOSD patients (632.2 pg/ml) and active SLE patients (771.6 pg/ml) compared with HC (1051.7 pg/ml, both p < 0.001). Plasma sRAGE levels were negatively correlated with AOSD activity scores (r = −0.320, p < 0.05). In comparison to AOSD patients with monocyclic pattern, significantly higher AGEs levels were observed in those with polycyclic or chronic articular pattern. With treatment, AGEs levels declined while sRAGE levels increased in parallel with the decrease in disease activity.

**Conclusion:**

The elevation of AGEs levels with concomitant decreased sRAGE levels in active AOSD patients, suggests their pathogenic role in AOSD.

## Background

Advanced glycation end products (AGEs) result from non-enzymatic glycation and glycoxidation of proteins, lipids, or nucleic acids [1,2]. Inflammation leads to oxidative stress and the consequent formation of reactive carbonyl compounds, which are partly transformed into AGEs [3,4]. The increased accumulation of AGEs has been reported in pathophysiological situations such as Alzheimer’s disease and inflammation [1-3]. The increased accumulation of AGEs has also been shown positively correlated with disease duration in systemic lupus erythematosus (SLE) [5-7]. These observations point to a pathogenic role of AGEs in inflammatory diseases.

Identified as a signal transduction receptor for AGEs [8,9], the receptor for AGEs (RAGE) consists of an N-terminal V-type Ig-like domain, two tandem C-type domains, a single transmembrane domain, and a short C-terminal intracellular cytoplasmic tail [8]. Accumulating evidence shows that RAGE plays critical role in inflammatory processes [10,11], and RAGE polymorphisms are associated with susceptibility and disease severity of lupus nephritis [12]. AGEs interact with both types of RAGE, namely full-length RAGE and C-truncated RAGE [13-15]. The interaction of AGEs with full-length RAGE induces activation of intracellular signaling and results in secretion of cytokines such as TNF-α, which plays a major role in inflammatory responses [13,14].

RAGE also exists in soluble form, termed soluble C-truncated RAGE (sRAGE), which lacks the transmembrane and cytosolic domains of the full-length RAGE. The sRAGE has the same AGEs-binding capacity as full-length RAGE [16], and may act as a “decoy” receptor by binding ligands (such as AGEs) and preventing them from interacting with full-length RAGE [17,18]. Therefore, decreased sRAGE levels may enhance full-length RAGE signaling and thereby augment inflammation. A previous study demonstrated that rheumatoid arthritis (RA) patients displayed lower sRAGE levels compared to healthy subjects and osteoarthritis patients [19]. Recent studies also observed low sRAGE levels in patients with active inflammatory diseases, such as Takayasu’s arteritis and SLE [20,21].

Adult onset Still’s disease (AOSD) is an inflammatory disorder, characterized by fever, rash, arthritis, multi-organs involvement, and increased acute phase reactants [22-26]. Although the aetiopathogenesis of AOSD remains unclear, cytokine-mediated inflammation may contribute to the development of this disease [27-29]. Therefore, we hypothesize that inflammatory responses in AOSD may be related to high AGEs levels and/or low sRAGE levels, which may lead to enhanced production of inflammatory mediators. However, there has been scanty data concerning circulating levels of AGEs and sRAGE in AOSD patients.

In this study, we examined plasma levels of AGEs and sRAGE in AOSD patients, SLE patients, and healthy subjects. Although AOSD has recently been considered as an autoinflammatory, instead of autoimmune, disease [30,31], we enrolled SLE patients as the disease control because both diseases share partial clinical manifestations, and the expression of AGEs or sRAGE has been observed in SLE [5,6,21]. The associations of AGEs and sRAGE levels with clinical characteristics, activity parameters, and disease courses were also investigated.

## Methods

### Patients

In this monocentric and prospective study, 52 patients with AOSD fulfilling the Yamaguchi criteria [32] were enrolled consecutively. Patients with infections, malignancies, or other rheumatic diseases were excluded. Disease activity of AOSD was assessed using a modified Pouchot score described by Rau et al. [33], and active AOSD was defined as a disease activity score of at least 3. All AOSD patients received corticosteroids and non-steroidal anti-inflammatory drugs (NSAIDs). Besides, the disease-modifying anti-rheumatic drugs (DMARDs) prescribed included hydroxychloroquine (42 patients), methotrexate (40 patients), sulfasalazine (28 patients), and azathioprine (12 patients). Defined as described in previous studies [34,35], one of three patterns of disease course was determined for each AOSD patient. Thirty-six patients fulfilling the 1997 revised criteria of the American College of Rheumatology (ACR) for SLE [36] were included as disease controls. Disease activity of SLE was determined by calculating SLE disease activity index (SLEDAI) [37], and active SLE was defined as a SLEDAI score of at least 6. Sixteen healthy volunteers who had no rheumatic disease were the normal controls. History of cigarette smoking, including past and present smoking habit, was taken and body mass index (BMI) was calculated as body weight in kilograms divided by the square of body height in meters. To decrease the influence of foods on AGEs levels, we advised the participants to avoid foods high in AGEs, such as donuts and dark-colored soda for at least one week before starting the investigation. Venous blood samples taken in the early morning were centrifuged at 1000 g for 10 min within 15 min of withdrawal, and all the plasma samples had been stored at −70°C until used simultaneously for the determination of levels of AGEs or sRAGE. To avoid the potential effects of hypoglycemic agents, statins, and angiotensin converting enzyme inhibitors (ACEI) on AGEs or sRAGE levels [38-40], subjects receiving these medications were excluded from this study. The ethics committee, the Institutional Review Board of Taichung Veterans General Hospital (CE11010), had approved the design of this study and the written consent was obtained according to the Declaration of Helsinki.

### Determination of plasma levels of AGEs and RAGE using ELISA

Plasma levels of total AGEs, which include N^ε^-Carboxymethyllysine (CML), pentosidine, and other AGE structures, were determined in all subjects using ELISA (Cell Biolabs Inc., San Diego, CA, USA) according to the manufacturer’s instructions. Plasma sRAGE levels were determined using another ELISA (R&D Systems, Inc., Minneapolis, MN, USA) which detects total sRAGE pool. The minimum detectable sRAGE level is 4.12 pg/ml. All assays were performed with both inter- and intra-assay coefficient of variation (CV) of less than 10%.

### Statistical analysis

The results are presented as the mean ± SD or median (interquartile range). Kruskal-Wallis test was used for between-group comparison of AGEs or sRAGE levels. When this test showed significant differences, the exact p-value was then determined using the Mann–Whitney U test. The correlation coefficient was obtained through the nonparametric Spearman’s rank correlation test. A logistic regression analysis was used to evaluate the effects of clinical characteristics and disease activity parameters on levels of AGEs or sRAGE in AOSD patients. Wilcoxon signed rank test was employed to compare the levels of AGEs and sRAGE during follow-up in AOSD patients after effective therapy. A probability of less than 0.05 was considered significant.

## Results

### Clinical characteristics of AOSD patients and SLE patients

Among the 30 patients with active AOSD, spiking fever (≥39°C), rash, arthritis, and lymphadenopathy were noted in 30 (100%), 25 (83.3%), 18 (60.0%), and 7 (23.3%) patients respectively. Twenty-five SLE patients had active disease (mean SLEDAI ± SD, 12.00 ± 1.11), eleven (30.6%) with renal involvement, and 11 had inactive disease (mean SLEDAI ± SD, 4.11 ± 0.63) at the time of investigation. There were no significant differences in age at onset, proportion of females, BMI, or proportion of past or present smoker between AOSD patients and SLE patients or healthy subjects (Table 1).

**Table 1:** **Demographic data and clinical characteristics of patients with adult-onset Still’s disease (AOSD), patients with systemic lupus erythematosus (SLE), and healthy controls (HC)#**

	**AOSD (n = 52)**	**SLE (n = 36)**	**HC (n = 16)**
Age at study entry (years)	37.1 ± 13.4	38.0 ± 12.1	32.5 ± 6.1
Proportion of females	37 (71.2%)	30 (83.3%)	10 (62.5%)
Serum creatinine (mg/dL)	0.84 ± 0.14	0.96 ± 0.92	0.89 ± 0.17
Body mass index (kg/m^2^)	22.6 ± 2.7	21.4 ± 1.8*	21.8 ± 1.7
AOSD activity scores	4.3 ± 2.0	NA	NA
CRP levels (normal <0.3 mg/dL)	2.66 ± 2.44	NA	NA
Ferritin levels (normal <280 μg/L)	1458 ± 4717	NA	NA
SLEDAI	NA	10.0 ± 1.0	NA
C3 (normal 84–160 mg/dL)	NA	86.9 ± 5.9	NA
C4 (normal 12–36 mg/dL)	NA	19.6 ± 2.1	NA
Anti-dsDNA (normal <92.7U/mL)	NA	251.4 ± 36.4	NA
Nephritis	0 (0.0%)	11 (30.6%)**	NA
Smoker (past)	10 (19.2%)	7 (19.4%)	3 (18.8%)
Smoker (present)	6 (11.5%)	5 (13.9%)	2 (12.5%)
Hypertension	4 (7.7%)	9 (25.0%)*^§^	0 (0.0%)

Data are presented as mean ± SD or number (percentage); NA: not applicable.

CRP: C-reactive protein; SLEDAI: SLE disease activity index; C3: complement 3; C4: complement 4; Anti-ds DNA: anti-double strand DNA antibody; Nephritis is defined by persistent proteinuria (>0.5 g/24 hours) or pathological examination of renal biopsy specimens showing lupus nephritis. *P < 0.05, **p < 0.001, vs. AOSD.

§p < 0.001, vs. Healthy controls.

### Plasma levels of AGEs in AOSD patients and SLE patients

**Figure 1:**
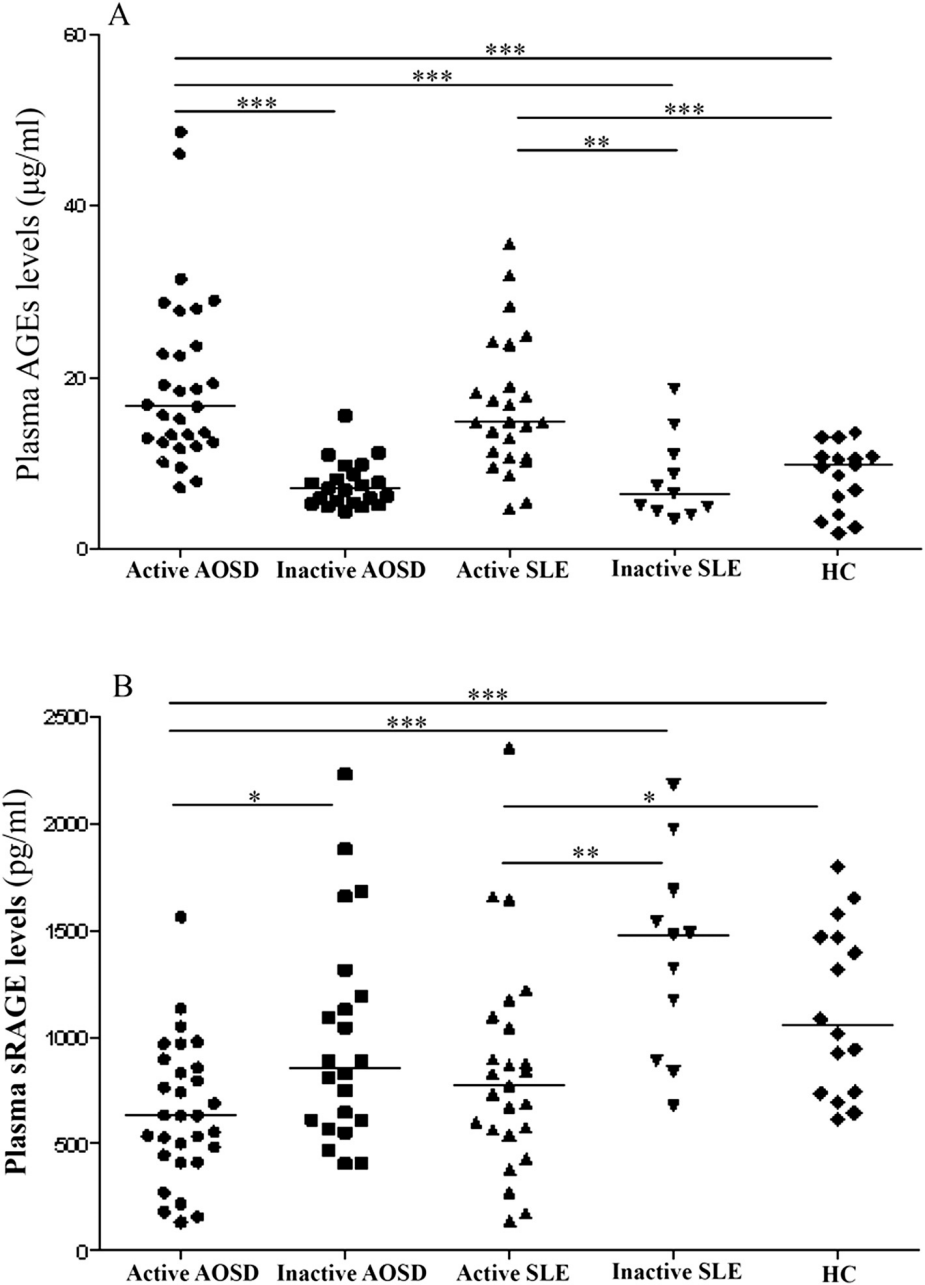
Comparison of plasma levels of **(A)** AGEs and **(B)** soluble RAGE (sRAGE) among 30 patients with active adult-onset Still’s disease (AOSD), 22 patients with inactive AOSD, 25 patients with active systemic lupus erythematosus (SLE), 11 patients with inactive SLE, and 16 healthy subjects (HC). The horizontal line indicates median value for each group. *p < 0.05, **p < 0.005, ***p < 0.001, was determined by Mann–Whitney U test.

As shown in Figure 1A and Table 2, significantly higher AGEs levels were observed in active AOSD patients and active SLE patients than the levels in healthy subjects. Significantly higher AGEs levels were also observed in active AOSD patients compared with inactive AOSD patients or inactive SLE patients. However, there was no significant difference in AGEs levels between active AOSD patients and active SLE patients.

**Table 2:** **Comparison of plasma AGEs levels and plasma sRAGE levels in 52 patients with adult-onset Still’s disease (AOSD), 36 patients with systemic lupus erythematosus (SLE), and 16 healthy subjects (HC)**

**AGEs and sRAGE, patient groups**	**Median (interquartile range)**
AGEs levels, μg/mL	
Active AOSD (n=30)	16.75 (12.45-24.66)**^##§§§^
Inactive AOSD (n=22)	7.02 (5.43-9.01)
Active SLE (n=25)	14.80 (10.68-21.42)**^##§§^
Inactive SLE (n=11)	6.49 (4.40-11.10)
HC (n=16)	9.80 (4.55-10.83)
sRAGE levels, pg/mL	
Active AOSD (n=30)	632.2 (437.8-866.9)**^#§§§^
Inactive AOSD (n=22)	858.9 (599.8-1222.2)^**§**^
Active SLE (n=25)	771.6 (553.0-1069.1)*^§§^
Inactive SLE (n=11)	1480.0 (892.1-1686.9)
HC (n=16)	1051.7 (738.2-1468.3)

AGEs: advanced glycation end products; sRAGE: soluble receptor for AGEs.

*p<0.05, **p<0.001, versus HC; ^#^p<0.05, ^##^p<0.001 versus inactive AOSD;

^§^p<0.05, ^§§^p<0.005, ^§§§^p<0.001, versus inactive SLE.

Mann-Whitney U test was used for between-group comparison of numerical variables.

### Plasma levels of sRAGE in AOSD patients and SLE patients

As shown in Figure 1B and Table 2, significantly lower sRAGE levels were observed in active AOSD patients and active SLE patients compared with healthy subjects. Active AOSD patients also had significantly lower sRAGE levels than inactive AOSD patients. However, there was no significant difference in sRAGE levels between active AOSD patients and active SLE patients.

### Correlation between disease activity parameters and AGEs levels or sRAGE levels

Plasma AGEs levels were positively correlated with disease activity parameters including clinical activity scores (r = 0.836, p < 0.001), serum ferritin levels (r = 0.372, p < 0.05), and CRP levels (r = 0.396, p < 0.005) in AOSD patients. Similarly, plasma AGEs levels were positively correlated with SLEDAI (r = 0.831, p < 0.001) in SLE patients. In contrast, plasma sRAGEs levels were inversely correlated with clinical activity scores (r = −0.320, p < 0.05) in AOSD patients, as well as SLEDAI (r = −0.483, p < 0.005) in SLE patients. In addition, there was an inverse correlation between AGEs levels and sRAGE levels in AOSD patients (r = −0.323, p < 0.05) and SLE patients (r = −0.454, p < 0.01).

### Logistic regression analysis

Using univariate regression analysis, AGEs levels were positively associated with the presence of anemia and disease activity parameters such as CRP levels and ferritin levels in AOSD patients (Table 3). Plasma sRAGE levels were negatively associated with the presence of anemia and leukocytosis in AOSD patients. Using univariate and multivariate analysis, AGEs levels were positively correlated with activity scores, while sRAGE levels were negatively correlated with activity scores in AOSD patients.

**Table 3:** **Logistic regression analysis of plasma AGEs levels and sRAGE levels in patients with adult-onset Still’s disease (AOSD)**

**AOSD patients (n = 52)**	**AGEs levels**			**sRAGE levels**		
	**B**	**Beta**	**p**	**B**	**Beta**	**p**
Univariate analysis						
Smoking	−1.00	−0.03	0.806	30.79	0.02	0.864
BMI	−1.01	−0.28	0.056	23.07	0.14	0.314
Leukocytosis	5.31	0.27	0.055	−313.40	−0.36	0.010
Anemia	6.89	0.30	0.032	−308.35	−0.30	0.031
CRP	2.21	0.55	0.000	−8.63	−0.05	0.734
Log(Ferritin)	10.18	0.58	0.000	−0.02	−0.16	0.244
Activity score	3.81	0.77	0.000	−85.88	−0.39	0.004
Multivariate analysis						
Activity score	2.63	0.53	0.000	−45.56	−0.21	0.062

AGEs: advanced glycation end products; sRAGE: soluble receptor for AGEs; BMI: body mass index; CRP: C-reactive protein.

Leukocytosis is defined as white cell count >10,000/cumm; anemia is defined as hemoglobin ≦11.3gm/dl.

### The differences in plasma levels of AGEs and sRAGE in AOSD patients with different patterns of disease course

**Figure 2:**
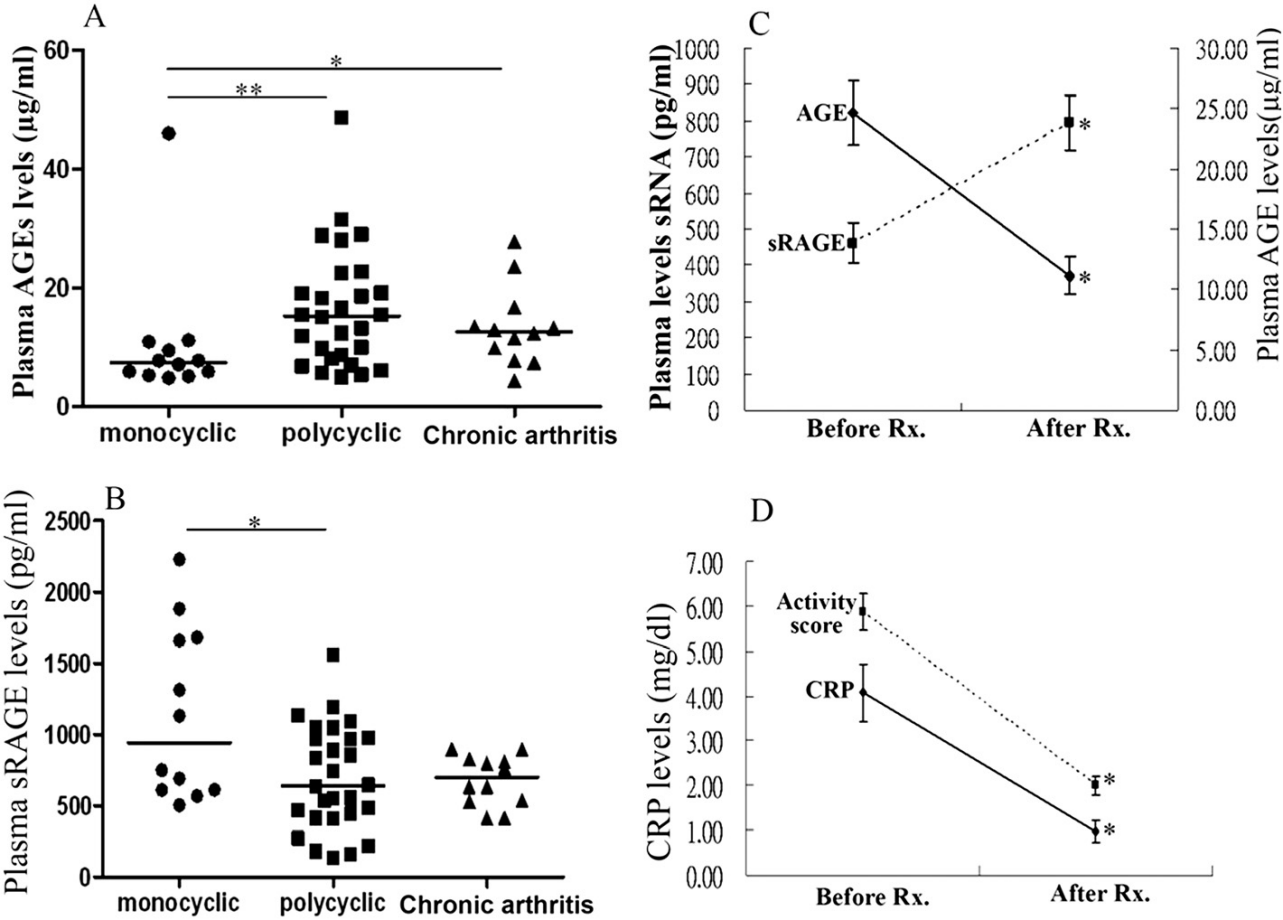
Comparison of plasma levels of **(A)** AGEs and **(B)** soluble RAGE (sRAGE) among AOSD patients with different patterns of disease courses, including 12 patients with monocyclic pattern, 28 patients with polycyclic pattern, and 12 patients with chronic arthritis. The horizontal line indicates median value for each group. *p < 0.05, **p < 0.005, was determined by Mann–Whitney U test. The changes in **(C)** plasma levels of AGEs as well as sRAGE and **(D)** disease activity parameters including clinical activity scores and C-reactive protein (CRP) levels in 16 AOSD patients after 6 months of treatment. Rx.: treatment. Data are presented as mean ± SEM. *p < 0.005, versus before treatment, determined by the Wilcoxon signed rank test.

Among all AOSD patients, 28 (53.8%) had polycyclic systemic pattern, 12 (23.1%) had monocyclic systemic pattern, and the remaining 12 (23.1%) had chronic articular pattern. As illustrated in Figure 2, significantly higher AGEs levels were observed in AOSD patients with polycyclic pattern or chronic articular pattern compared with those with monocyclic pattern. In contrast, significantly lower sRAGE levels were demonstrated in AOSD patients with polycyclic pattern than those in patients with monocyclic pattern.

### Changes in plasma levels of AGEs and sRAGE in AOSD patients after therapy

Sixteen AOSD patients were available for examination both in the active phase and in the inactive phase after 6 months of treatment. As shown in Figure 2C and D, AGEs levels significantly declined (mean ± SEM, 24.6 ± 2.7 pg/mL vs. 11.2 ± 1.6 pg/mL, p < 0.001) while sRAGE levels increased (460.5 ± 55.8 pg/mL vs. 794.9 ± 77.0 pg/mL, p < 0.001) significantly, paralleling the decreases in clinical activity scores (5.88 ± 0.41 vs. 2.00 ± 0.20, p < 0.001) and CRP levels (4.07 ± 0.63 pg/ml vs. 0.97 ± 0.26 pg/ml, p < 0.001) in AOSD patients after 6-month therapy.

## Discussion

This is the first study to examine plasma levels of AGEs and sRAGE in AOSD patients, and to characterize their association with activity parameters of this disease. Plasma AGEs levels were significantly higher in active AOSD patients compared with inactive AOSD patients or healthy subjects, with the levels positively correlated with activity parameters. In contrast, plasma sRAGE levels were significantly lower in active AOSD patients compared with inactive AOSD patients or healthy subjects, with their levels negatively correlated with activity parameters. Moreover, the decrease in AGEs levels and an increase in sRAGE levels paralleled disease remission. These observations indicate that overproduction of AGEs and a reverse regulation of sRAGE may be involved in AOSD pathogenesis, and may act as activity indicators of this disease.

RAGE, the most characterized receptor of AGEs, appears to play an important role in inflammatory responses [9-12]. Soluble RAGE (sRAGE), the extracellular domain of RAGE, is generated by two distinct mechanisms, the splice variant [endogenous secretory RAGE (esRAGE)] secretion and cleavage of membrane-bound RAGE (cRAGE) through metalloproteinase [15,16]. Both esRAGE and cRAGE are functionally equivalent and interact with the same RAGE ligands. The sRAGE acts as a decoy receptor, and effectively blocks the binding of AGEs or other ligands to cell membrane-bound RAGE [17,18], which may explain the inverse correlation between sRAGE levels and AGEs levels in AOSD patients. The reduction of sRAGE levels may also represent an inadequate protective response in inflammatory diseases. Likewise, circulating sRAGE levels have been shown to be decreased in patients with RA [19], Takayasu’s arteritis [20], SLE [21], and Still’s disease [41]. In agreement with previous findings that decreased sRAGE levels were negatively correlated with disease activity in patients with Still’s disease [41], we similarly demonstrated significantly lower sRAGE levels in active AOSD patients than those in quiescent AOSD patients, with an inverse correlation between plasma sRAGE levels and disease activity. Therefore, plasma sRAGE level may serve as a biomarker and negative regulator of inflammation [42], and the administration of recombinant sRAGE has been of therapeutic use in inflammatory murine models [43,44].

As with other previous findings [5,6,21] and similar to AOSD patients, our SLE patients also had increased AGEs levels which were positively correlated with SLEDAI. On the other hand, active SLE patients had decreased plasma sRAGE levels negatively correlated with disease activity, substantiating the findings that treatment with sRAGE significantly improved nephritis and histologic renal damage in lupus-prone (NZB/NZW) mice [45].

There are two possible mechanisms that may explain the low sRAGE levels in our active AOSD patients. One is that plasma sRAGE levels may be modulated by its binding with inflammatory ligands, thereby promoting consumption of sRAGE [46]. Compatible with this hypothesis, an elevated AGEs levels may increase the binding and consumption of sRAGE in this study. The other is that the reduced production of endogenous secretory sRAGE may not be sufficient as a decoy receptor for binding inflammatory ligands and therefore there is weakened protection against the inflammatory response. The inadequate levels of sRAGE might amplify inflammatory responses by increasing the engagement of accumulated AGEs on cell-bound RAGE [19].

Interestingly, a logistic regression analysis demonstrated that plasma AGEs levels were positively associated with the presence of leukocytosis, whereas sRAGE levels showed negative association. Because cell-bound RAGE may function as a counter-receptor for leukocyte integrin Mac-1 and was directly involved in leukocyte recruitment, sRAGE could act as a potential inhibitor of leukocyte recruitment [47]. In addition, we found that the presence of anemia in AOSD patients was positively associated with AGEs levels, but negatively associated with sRAGE levels, which resonates with the findings that plasma AGEs, a marker of oxidative stress, was a predictor of anemia in older community-dwelling adults [48].

The disease courses of AOSD patients may vary considerably. Those with polycyclic disease pattern exhibit relapsing episodes with systemic inflammation superimposed on chronic low-grade inflammation. It is interesting to note that significantly higher AGEs levels were observed in AOSD patients with polycyclic pattern or chronic arthritis compared with those with monocyclic pattern. Cumulative inflammation may contribute to the increased AGEs levels in AOSD patients with polycyclic pattern or chronic arthritis, as with the enhanced AGEs levels positively related to the disease duration of SLE [5], and the elevation of AGEs levels in RA patients [3]. In contrast, plasma sRAGE levels were lower in AOSD patients with polycyclic pattern than in those with monocyclic pattern. Thus, we hypothesize that the dysregulated inflammation in patients with polycyclic pattern may persist via insufficient inhibition by sRAGE.

Longitudinal follow-up of our AOSD patients showed that AGEs levels declined while sRAGE levels increased with therapy, paralleling the reduction of disease activity. The change in sRAGE levels after 6-month therapy was similar to a recent finding that sRAGE levels increased after long-term (more than one month) treatment [21], and further supports the potential for AGEs or sRAGE as a biomarker of disease activity [42].

There were some limitations in our study. Because AGEs represent a heterogeneous group of compounds, future studies are needed to determine the major compound which is related to AOSD. The sRAGE may act as a decoy receptor; however, we still did not elucidate the functional role of sRAGE in AOSD. Our preliminary results await further substantiation by *ex vivo* studies which will determine interleukin-1 secretion by subjects’ monocytes treated with AGEs or sRAGE.

## Conclusions

In conclusion, increased AGEs as well as decreased sRAGE, which were significantly correlated with disease activity parameters, may be involved in AOSD pathogenesis. We provide the first evidence showing that plasma levels of AGEs and sRAGE may be linked with disease outcome in AOSD. The results could be of translational interest, and may potentially lead to the discovery of novel activity biomarkers [42] or promising therapeutic targets such as sRAGE, which has been used to protect against colonic inflammation or autoimmune encephalitis [43,44].

### Key messages

1. Elevated AGEs levels and decreased sRAGE levels were observed in both AOSD and SLE.
2. Associations of AGEs and sRAGE levels with disease activity indicate their involvement in pathogenesis.

## Abbreviations

ACEI: Angiotensin converting enzyme inhibitors
ACR: American College of Rheumatology
AGEs: Advanced glycation end products
AOSD: Adult onset Still’s disease
BMI: Body mass index
cRAGE: Cleavage of membrane-bound RAGE
CV: Coefficient of variation
DMARDs: Disease-modifying anti-rheumatic drugs
esRAGE: Endogenous secretory RAGE
NSAIDs: Non-steroidal anti-inflammatory drugs
RAGE: Receptor for AGEs
SLE: Systemic lupus erythematosus
SLEDAI: SLE disease activity index
sRAGE: Soluble C-truncated RAGE
